# Smallpox and Vaccination

**Published:** 1901-02

**Authors:** 


**A Monthly Review of Medicine and Surgery.**

*EDITOR:*

WILLIAM WARREN POTTER, M. D.

All communications, whether of a literary or business nature, books for review and exchanges should be addressed to the editor: 284 FRANKLIN STREET, BUFFALO, N. Y.

VOL. XL.—LVI. FEBRUARY, 1901. No. 7.

### SMALLPOX AND VACCINATION.

HEALTH Commissioner Wende has issued a proclamation concerning vaccination and the prevalence of smallpox in various parts of the United States, which is timely and to the point. He called attention to the fact that the disease was prevalent in states contiguous to Buffalo, and that safety lay in proper and immediate vaccination, especially in schools and factories and other places where numbers of people are gathered together. The Health Commissioner's warning note is in no sense an alarm or a panic breeder. It is a quiet, conservative statement of fact. Its only fault, if fault it has, is its conservatism. This may be justified in view of the fact that the Pan-American Exposition is about to open its doors, yet the fact remains that smallpox does exist in almost epidemic form in many localities and that it will in all probability be brought into Buffalo, if not before then, by some of the Pan-American visitors. To meet this possibility, wholesale vaccination is an absolute necessity. Of course, no power exists to compel vaccination in every case, yet the majority of people are sensible-minded and will submit.

Naturally, that brave and devoted band of fanatics, who hang on the honeyed words which flow from Mother Eddy's inspired lips, will hold out and decline to entertain the "belief" that there is such a disease as smallpox. So will some of the followers of that eminent and spooky divine who lately discoursed upon the subject. Of course, we must meet and deal with cranks of all descriptions; natural cranks, fool cranks and imbecile cranks, like the anti-vaccinationists, anti-vivisectionists and anti-humanity howlers, who delve into the depths of the dusty mysteries of spook-chasing and Eddyite beliefs.

The Health Commissioner has done his duty thus far. He cannot be held to blame should smallpox make its appearance in Buffalo. It is impossible to give definitely the smallpox figures as they stand at this writing, but an adequate idea may be had when it is known that there are approximately 8,000 cases now recorded as compared with 1,700 of a year ago. This in itself is a serious increase, but the real seriousness of the situation becomes apparent when it is reported that smallpox is on the increase in New York city, and that it is in Ohio, Pennsylvania and Canada. The district physicians have held a meeting to consider the question of vaccinating in the public and private schools. Leaving outside the ethical question of district health physicians doing charity vaccinating among school children, the majority of whose parents are perfectly able to pay a family physician for the operation, the fact that school children should be vaccinated and that promptly is apparent. If they are not protected they should not be admitted to the schools. Let the fanatics pray for deliverance from an epidemic, but in the meantime let us vaccinate.

---

THE second free public bathhouse in Buffalo was thrown open for use January 2, 1901. It is located at the corner of Stanislaus Street and Woltz Avenue. The first day it was in use 350 persons availed themselves of its privileges. This bathhouse is in the center of the Polish district and must prove of great benefit to the residents of the eastern portion of the city. The new bathhouse is larger than the first one erected, is a stone and frame structure, and is equipped with every convenience.

---

DR. SAMUEL FLOERSHEIM, of 218 East 46th Street, New York City, announces his intention to publish a second paper on the use of the suprarenal capsule in organic heart disease. He requests the readers of this journal to send him the reports of their cases as follows: (1) the condition of the heart and pulse rate; (2) the effect on the heart and pulse, and pulse rate within ten minutes after the suprarenal powder; three grains, is chewed and swallowed without water, by the patient.

---

THE University of Pennsylvania has made a move, which it is believed is the beginning of the revolutionising of medical instruction. It is the establishment of a complete curriculum of medicine. The branches are so arranged, that each year's work leads up to the succeeding, and above all, the same conditions for entering the medical department are exacted from candidates, as those which cover other departments. Thus, the degree of M.D. is a college degree. This excludes that large proportion of inadequately educated students, who hitherto have constituted the mass of incompetent material which has so seriously burdened the profession.

---

THE New York Academy of Medicine through its committee on library, has recently made its annual report. From this we note that the number of books in the library, November 31, 1900, was 89,000 volumes, including duplicates, of which there are 36,105 volumes. The books added during the year, not including duplicates, were 3,649 volumes. In the circulating department, 971 books and 832 journals were issued to 194 readers. 11,520 readers have registered during the year in the reading room, and probably many others have used the library, but not registered. The library is growing rapidly, the additions annually for the last four years being at least 3,500 volumes a year—in 1898, when the library of the New York Hospital was donated to the Academy 7,000 volumes—without including duplicates. 991 journals in many languages are on file, and add 1,135 bound volumes this year to the shelves. Including the duplicates the natural growth annually amounts to about 5,000 volumes.

---

DR. GEORGE M. GOULD, the accomplished editor of the *Philadelphia Medical Journal*, has been dischaiged from his office by the Board of Trustees without notice, complaint or criticism of his editorial management of that journal. This couise, to say the least, is unusual and according to all precedent may be justly characterised as unfair, no matter what reasons may have led to such summary procedure. Dr. Gould has made a good journal and his readers will be sorry to miss his trenchant pen from its pages.

It is rumored that he will establish another journal, provided he receives substantial encouragement from the medical profession. We bespeak success for the enterprise should it materialise.

---

A PRESS dispatch informs us that experiments lately made by E. H. Fairchild in the operating rooms of the Albany Hospital, have proved that it is possible to get a series of photographs of a surgical operation, and thus to provide lantern slides as a basis for surgical instruction. Operations by Drs. Albert Vander Veer and Willis G. Macdonald have been successfully photographed in series, each stage in the operation being represented by a photograph taken instantaneously and without in anyway interfering with the operation.

Students of surgery find it difficult to secure satisfactory instructions, because during an operation the surgeons are too much absorbed to lecture, and afterward the opportunity is gone. By means of a special camera a series of photographs can be taken, showing all that any student can see, and with these as a basis the surgeon can give an illustrated lecture. A course of illustrated lectures explanatory of all the common operations, and also of the exceptional and remarkable operations that come so rarely as to make it impossible to secure clinical material for every class, can be built up around such a group of pictures.

---

INFLUENZA has invaded almost every northern state to a degree that in many localities has seriously embarrassed business. Buffalo has suffered its full share from the disease which, however, has not been markedly fatal except with the aged, or from those previously suffering from some systemic disease. Moist and sodden weather, such as has prevailed for a considerble period during the winter, furnishes a favorable medium for the propagation and transmission of the germs. At such a time it behooves everybody to be cautious about exposure and to be particular with reference to warm underwear. Mouth breathing, too, which is so very common, should be absolutely interdicted in the open air.

---

THE new State Pharmacy Law of New York went into effect the beginning of the year, when the three boards and three laws formerly existing, were replaced by one board and one law having jurisdiction over the whole state. This is an advantage over the old regulations and while the new law is open to criticism in some particulars, it is regarded as being much superior to the confusion of the three boards. One of the important provisions of the law is the annual registration under restricted conditions, not of the pharmacists themselves, but of every pharmacy store, dispensary or place where medicines are sold.

The president of the state board is Mr. R. K. Smither, of Buffalo, and Mr. Sidney Faber, of New York, is secretary and treasurer. Each of the three divisions have their president and secretary. Of the western section, Mr. M. A. Palmer, of Olean, is president, and Mr. George Reimann, of Buffalo, is secretary.

Dr. G. Frank Lydston, of Chicago, is well known to the profession as an able teacher, a clever writer and a successful practitioner. He has written one of the best works on genito-urinary diseases of recent days, and he is a frequent contributor to medical periodicals of excellent professional papers.

But there is another field in which Dr. Lydston is rapidly achieving fame—namely, in general literature. He not only tells a story well, but he writes one with grace and makes it entertaining. His "Over the Hookah" has enabled his readers to beguile many an hour and his numerous sketches, scattered here and there, are sources of delight to those familiar with them. We are prompted to these remarks because he has lately sent out to the world an entertaining volume entitled, Panama and the Sierras; a Doctor's Wander Days. It is a description of scenes witnessed and experiences met by the author during a journey across the Isthmus of Panama. It is profusely illustrated from photographs taken by the author and is published by the Riverton Press, Chicago.

